# Individual, community and societal correlates of insecticide treated net use among pregnant women in sub-Saharan Africa: a multi-level analysis

**DOI:** 10.1186/s12889-021-11635-6

**Published:** 2021-08-26

**Authors:** Edward Kwabena Ameyaw

**Affiliations:** 1 School of Public Health, Faculty of Health, University of Technology Sydney, Sydney, Australia; 2 L & E Research Consult, Wa, Upper West Region Ghana

**Keywords:** Insecticide treated net, Pregnancy, Pregnant women, Sub-Saharan Africa, Public health

## Abstract

**Background:**

Malaria in pregnancy is a crucial public health concern due to the enormous risk it poses to maternal and newborn health. The World Health Organisation therefore recommends insecticide-treated net (ITN) for pregnant women. The world over, sub-Saharan Africa bears the highest prevalence of malaria and its associated complications. This study investigated the individual, community and society level factors associated with ITN use among pregnant women in sub-Saharan Africa.

**Methods:**

The study was conducted with Demographic and Health Survey data of 21 sub-Saharan African countries. A total of 17,731 pregnant women who possessed ITN participated in the study. Descriptive computation of ITN use by survey country and socio-demographic characteristics was conducted. Further, five multi-level binary logistic regression models were fitted with MLwiN 3.05 package in STATA. The Markov Chain Monte Carlo (MCMC) estimation procedure was used in estimating the parameters whilst the Bayesian Deviance Information Criterion was used for the model fitness test.

**Results:**

On average, 74.2% pregnant women in SSA used ITN. The highest prevalence of ITN use occurred in Mali (83.7%) whilst the least usage occurred in Namibia (7%). Women aged 30–34 were more likely to use ITN compared with those aged 45–49 [aOR = 1.14; Crl = 1.07–1.50]. Poorest women were less probable to use ITN relative to richest women [aOR = 0.79; Crl = 0.70–0.89]. Compared to women who did not want their pregnancies at all, women who wanted their pregnancies [aOR = 1.06; Crl = 1.04–1.19] were more probable to use ITN. Women in male-headed households had higher likelihood of ITN use compared to those from female-headed households [aOR = 1.28; Crl = 1.19–1.39]. On the whole, 38.1% variation in ITN use was attributable to societal level factors whilst 20.9% variation was attributable to community level factors.

**Conclusion:**

The study has revealed that in addition to individual level factors, community and society level factors affect ITN use in SSA. In as much as the study points towards the need to incorporate community and societal variations in ITN interventions, active involvement of men can yield better outcome for ITN utilisation interventions in SSA.

## Background

Malaria accounts for 900,000 deaths every year with most of these deaths transpiring in sub-Saharan Africa (SSA). In 2018, Africa borne the highest proportion of malaria morbidity with 213 million cases (93%) [1]. The sub-Saharan African countries highly plagued by malaria in pregnancy and its related consequences have some utmost concentration of other risks for unhealthy pregnancies, unhealthy newborns and children [1]. The World Health Organization (WHO) stresses universal access to malaria prevention tools as promising pathway toward malaria elimination especially among pregnant women [2].

Malaria infection during pregnancy is a prime public health concern, due to the enormous risks to maternal and perinatal mortality and morbidity [1]. Malaria in pregnancy can cause miscarriage, premature delivery, perinatal death, low birth weight and congenital infections [3]. Even though the pathway between malaria and pregnancy is not fully understood, pregnant women generally have declined immune response and as such limited in combating malaria [3]. For pregnant women, the malaria parasites impound and replicate within the placenta. Malaria symptoms and complications in pregnancy vary based on the intensity of transmission within geographical location and women’s level of immunity [4]. In SSA for instance where 29% overall exposure to malaria in pregnancy from moderate to high transmission exists, the 2019 World Malaria report acknowledged notable geographical variations [1]. The highest prevalence of malaria exposure in pregnancy occurred in Western and Central Africa (35% each) whilst 20% of prevalence of exposure occurred in Eastern and Southern Africa in 2018 [1].

The WHO has recommended the use of insecticide-treated nets (ITNs) by pregnant women. Although, ITN use is not beyond the recommendations of WHO [2], it is one of the most cost-effective tools available to control malaria all over the world [1]. ITN is a treated, safe and effective net for reducing human contact with mosquitoes. The distribution of ITN in endemic locations has contributed substantially towards reduction in malaria episodes, severe disease, and malaria-related deaths [1]. The ITN contributes to 48–50% reduction in malaria episodes [5] and has shown statistically significant decline in low birth weight and fetal loss risks [6]. Concerted global efforts have also been directed to mitigating malaria particularly among pregnant women. For instance, the WHO and the Roll Back Malaria (RBM) Partnership to End Malaria initiated the high burden to high impact (HBHI) strategy to ensure that the 11 leading malaria endemic countries achieve the 2025 Global Technical Strategy (GTS) milestone [2, 7]. All the 11 countries are within SSA except India. An estimated funding of US$ 9.4 billion had been disbursed to these countries between 2010 and 2018 [1]. However, the current prevalence of malaria in pregnancy within SSA suggests that these commitments have not made much gain considering the proportion of women who stand a chance of malaria exposure in pregnancy. Thus, over 25 million pregnant women in sub-Saharan Africa are at risk of *P. falciparum* infection [8].

Previous studies have focused on country level predictors of ITN use [9–12]. Reported significant predictors of ITN use by the aforementioned studies include occupation, educational attainment, antenatal care (ANC) attendance and literacy. Although some of these studies investigated ITN use at different levels (e.g. individual and community), the studies were limited to a few specific SSA countries. ITN ownership rate in SSA ranges between 3 and 80%, however 17% ITN use has been reported among pregnant women [13–15]. In order to end malaria in pregnancy in SSA, it is expedient to quantify the current ITN use among pregnant women in SSA, taking cognisance of individual, community, and societal contexts as little is known about how these factors mediate ITN use in the sub-region. Subsequently, this study determined the individual, community and societal factors that are associated with ITN use among pregnant women in SSA.

## Methods

### Study setting

According to the United Nations, sub-Saharan Africa consists of all African countries and territories that are partially or fully south of the Sahara [16]. Geographically and ethnoculturally, SSA is the area or region of the African continent that lies south of the Sahara. Although the geoscheme of the United Nations excludes Sudan from its definition of SSA, the African Union’s definition captures Sudan but excludes Mauritania instead [16]. It is worth knowing that Demographic and Health Surveys (DHSs) are not conducted in all SSA countries.

### Data source and sample

The data supporting this study emerged from the latest Demographic and Health Surveys (DHS) conducted between January 1, 2010 and December 31, 2018 in 21 SSA countries. DHS surveys of these countries were used due to availability of ITN and all essential variables that were required for the study. These surveys are conducted through the DHS Program in collaboration with participating countries globally. These surveys are carried out in low and middle-income countries at five-year interval [17]. Since commencement in 1984, the focus of the survey has been on essential maternal and child health factors including malaria prevention, intermittent preventive treatment in pregnancy (IPTp) among other factors [17, 18]. The women’s files from included countries were used. A common sampling procedure is adopted for all the surveys. Thus, stratified two-stage sampling approach is followed to recruit participants during the surveys. The primary stage involves the selection of clusters (i.e., enumeration areas [EAs]). The second stage constitutes a systematic household sampling in the selected EAs. The current study was restricted to 17,731 women who reported that they were pregnant and possessed ITN during the survey. Comprehensive sampling approaches of DHS have been articulated in the reports of the respective countries [19–38]. Included countries and their respective samples are summarised in Table 1.

**Table 1:** Sample size distribution by country and survey year

Survey countries	Survey year	Weighted sample (n)	Weighted Percentage (%)
**Central Africa**			
Congo-DR	2014	1563	8.8
Gabon	2012	389	2.2
Malawi	2015–2016	1181	6.7
**West Africa**			
Benin	2017–2018	1030	5.8
Gambia	2013	632	3.6
Ghana	2014	477	2.7
Guinea	2018	574	3.2
Liberia	2013	421	2.4
Nigeria	2018	2909	16.4
Niger	2012	1139	6.4
Mali	2018	1018	5.7
Sierra Leone	2013	815	4.6
Togo	2013–2014	458	2.6
**East Africa**			
Burundi	2016–2017	774	4.4
Kenya	2014	591	3.3
Mozambique	2015	371	2.1
Tanzania	2015–2016	764	4.3
Uganda	2016	1352	7.6
Zambia	2018	842	4.7
Zimbabwe	2015	327	1.8
**Southern Africa**			
Namibia	2013	104	0.6
**Total**	**–**	**17,731**	**100**

DHS conducted in 21 SSA countries: 2012–2018

### Variables

#### Outcome variable

The principal outcome variable for the study was ITN utilisation among pregnant women. All women who participated in the survey responded to whether they slept under ITN the night before the interview with “yes = 1” or “no = 0” responses. Based on recommendation by the WHO’s Roll Back Malaria Monitoring and Evaluation Reference Group (MERG) [39] and previous studies [40, 41], all pregnant women who choose “yes” were deemed as utilising ITN whilst those who responded “no” were labelled as not using ITN.

#### Explanatory variables

Thirteen explanatory variables layered hierarchically under individual, community and society levels were included in this study. In line with literature [42, 43], 10 of the variables were fitted under the individual level factors. Individual level factors consist of a range of factors that identifies an individual or her key characteristics. These are age (15–19, 20–24, 25–29, 30–34,35–39, 40–44, 45–49), education (no education, primary, secondary, higher), marital status (married, cohabiting), healthcare decision making (alone, with partner, husband/partner alone, other person), wanted current pregnancy (wanted, later, not at all) and health insurance subscription (no, yes). Other individual level factors were frequency of listening to radio (not at all, less than a week, at least once a week, almost every day), frequency of watching television (not at all, less than a week, at least once a week, almost every day) and frequency of reading newspaper (not at all, less than a week, at least once a week, almost every day).

At the community level, residence (urban, rural), wealth quintile (poorest, poorer, middle, richer, richest), sex of family head (male, female) [44], community literacy level (low, medium and high), and community socio-economic status (low, medium and high) were considered. The term “community level factors” comprise a range of factors that can influence health, safety, wellbeing and participation in physical activity [45]. Community-level socio-economic status was generated by aggregating the individual-level data into cluster. Thus, it was derived from the proportion of poor, unemployed and illiterate people in the community [43]. A standardized score having mean of 0 and standard deviation 1 was generated; with higher scores indicative of the lower socioeconomic position (SEP). Resultant scores were apportioned into tertiles to permit nonlinear effects and offer results that are readily interpretable in the policy arena [43].

Following the same procedure, community literacy level was derived from the proportion of women who could read and write in the community. The specific locations of the countries constituted the societal factor. Due to the strong association between survival of mosquitos or incidence of malaria and geographical/climatic factors [46–48], the countries were categorised based on their specific location within Africa as defined by the United Nations [49]. These are Western (Benin, Burkina Faso, Ghana, Guinea, Liberia, Mali, Niger, Sierra Leone, Togo), Eastern (Burundi, Kenya, Malawi, Mozambique, Tanzania, Uganda, Zambia, Zimbabwe), Central (Congo DR, Gabon, Guinea) and Southern (Namibia).

### Analysis and modelling approach

**Fig. 1:**
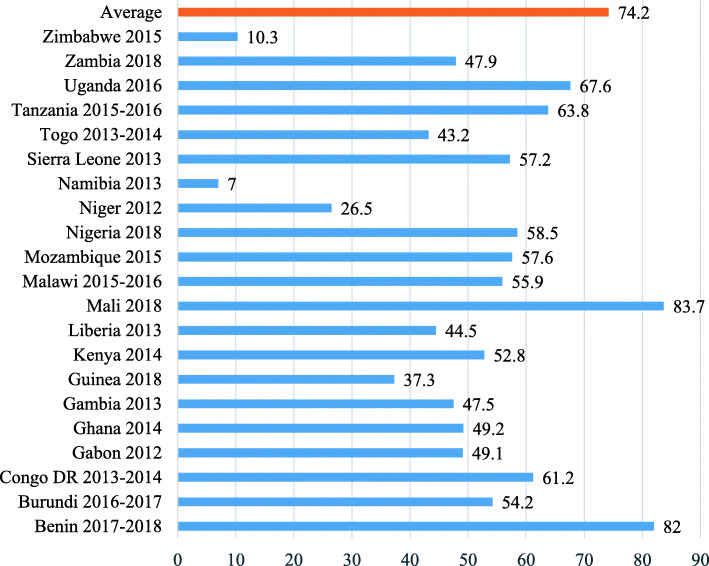
Prevalence of ITN utilisation in SSA

The first step of the analyses was the computation of the prevalence of ITN by survey country (see Fig. 1). Datasets of all the 21 countries were then appended subsequently. Socio-demographic characteristics of the women were weighted and computed. Prevalence of pregnant women who utilised ITN were calculated with respect to the individual, community and society level characteristics. The association between each of the variables and ITN utilisation was assessed with chi-square at 95% significance threshold. Due to the hierarchical structure of the dataset, multilevel modelling was applied using the MLwiN package version 3.05 [50].

A three-level level multilevel logistic regression analysis was carried out and five models were fitted to investigate the effect of individual, community and society level factors on ITN utilisation. In order to explore the complete variance in ITN utilisation across societies, no independent variable was included in the first model (empty model) as illustrated in Table 3. The second model accounted for fixed effect at the individual level and random effect. The third model considered the fixed effect at the community level in addition to cluster-level random effects at the community and societal level. The fourth model constitutes the fixed effect at the societal level together with cluster random effects at the community and society level. In the complete and final model (model 5), fixed effect at the individual, community and society level in addition to cluster random effect at these three levels were computed (Table 3).

Fixed effect results were presented as odds ratios (ORs) and adjusted odds ratios (aOR) with their respective 95% credible intervals (95% Crl). The credible interval was premised on Bayesian statistical approach such that the likelihood distribution for measures of association (odds ratios (ORs) or adjusted odds ratios (aORs), are obtained whilst 95% credible (95% Crl) intervals are permitted for the purpose of summarising as opposed to 95% confidence intervals (95% CI). With 95% Crl, there is a chance of the parameter assuming a value in the range specified [51]. The results for the random effects were computed as variance partition coefficient (VPC), also known as intraclass correlation coefficient (ICC), and median odds ratio (MOR) [52, 53]. Whilst VPC measures the extent of variance in the likelihood of ITN utilisation that is attributable to community and society levels, the MOR measures the community or societal variance in terms of odds radio and computes the likelihood of ITN utilisation that is attributable to community and society level characteristics. Normative categories were considered for the reference groups. Prior to modelling, collinearity was checked for all the explanatory variables with the variance inflation factor [54] and the outcome indicated the absence of multicollinearity (mean VIF = 1.27; maximum = 1.70; minimum = 1.03).

#### Ethical consideration

The DHS Program reports common ethical procedures for all the 21 countries. Ethical approval for the DHS are obtained from the ethics committee of ORC Macro Inc. and collaborating institutions of the survey countries, usually the ministries of health of the respective countries Additionally, the Inner-City Fund (ICF) International maintains that the procedures of DHS do not contradict the regulations for the respect of human subjects as required by the U.S. Department of Health and Human Services. Since the data sets are publicly available, no additional ethical requirements were needed for this study. Detailed information about the ethical protocols have been documented elsewhere [55].

## Results

### Prevalence of ITN use among pregnant women in SSA

As shown in Fig. 1, 74.2% pregnant women in SSA used ITN on the average. Across countries, the highest usage occurred in Mali (83.7%) and the least prevalence of ITN use occurred in Namibia (7%).

### Socio-demographic characteristics and ITN utilisation among pregnant women

Table 2 presents ITN utilisation with respect to socio-demographic characteristics of study participants. ITN use was highly reported by women aged 20–24 (76.9%) and women having primary education (76.7%) and poorer women (77.2%). Most cohabiting women used ITN (77.7%) just as women who decided on their healthcare with their husbands/partners (75.3%). At least seven out of 10 of the women who did not want their pregnancies at all utilised ITN (75.5%) and this was similar to those who were subscribed to health insurance and used ITN (74.5%). Most women who did not listen to radio at all (76.2%), those who did not watch T.V. at all (75.5%) and those who did not read newspaper at all (74.7%) reported that they utilised ITN. Similarly, most poor women (77.2%) and women in rural residence utilised ITN (74.8%) as well as those in male-headed households (75.1%). ITN use stood at 75.5% among communities with low literacy. Similarly, 73.9% ITN usage was observed among communities with high socio-economic status.

**Table 2:** Socio-demographic characteristics and ITN utilisation among pregnant women (*n* = 17,731)

Variable	Weighted N	Weighted Percentage (%)	Slept under ITN		χ^**2**^ (df), ***p***-value
			Yes (%)	No (%)	
***Individual level***					
**Age, years**			13,163(74.2)	4568(25.8)	55.091 (6), *p* < 0.001
15–19	2249	13.0	1553(69.4)	684(30.6)	
20–24	4428	25.0	3412(76.9)	1022(23.1)	
25–29	4637	26.1	3526(76.4)	1086(23.6)	
30–34	3484	19.6	2603(75.0)	866(25.0)	
35–39	2068	11.6	1534(73.7)	546(26.3)	
40–44	698	3.9	536(73.0)	198(27)	
45–49	165	0.93	119(72.1)	46(27.9)	
**Education**					15.946 (3), *p* < 0.01
No education	7111	40.1	5244(74.6)	1789(25.4)	
Primary	5675	32.0	4449(76.7)	1353(23.3)	
Secondary	4285	24.2	3127(73.4)	1136(26.6)	
Higher	659	3.7	463(73.1)	170(26.9)	
**Marital status**					25.295(1), *p* < 0.001
Married	14,630	82.5	10,752(73.5)	3878(26.5)	
Cohabiting	3101	17.5	2411(77.7)	690(22.3)	
**Health decision making**					52.078. (3), *p* < 0.001
Alone	2284	12.9	1579(69.1)	706(30.9)	
With Partner	6681	37.7	5033(75.3)	1648(24.7)	
Husband/Partner Alone	8628	48.7	6468(75.0)	2160(25.0)	
Other person	137	0.8	84(61.2)	53(38.8)	
**Wanted current pregnancy**					1.179 (2), *p* = 0.555
Wanted	13,124	74.0	9801(74.7)	3323(25.3)	
Later	3571	20.1	2580(72.3)	990(27.7)	
Not at all	1036	5.8	782(75.5)	254(24.5)	
**Health insurance**					2.644 (1), *p* = 0.103
No	16,343	92.9	12,242(74.5)	4192(25.5)	
Yes	1298	7.3	921(71.0)	375(29.0)	
**Frequency of listening to radio**					38.592(2), *p* < 0.001
Not at all	7230	40.8	5506(76.2)	1724(23.8)	
Less than once a week	3685	20.7	2672(72.5)	1013(27.5)	
At least once a week	6489	36.6	4800(74.0)	1688(26.0)	
Almost every day	327	1.8	184(56.4)	142(43.6)	
**Frequency of watching T.V**					36.049 (3), *p* < 0.001
Not at all	11,678	65.9	8820(75.5)	2857(24.5)	
Less than once a week	2114	11.9	1525(72.1)	589(27.9)	
At least once a week	3427	19.3	2505(73.1)	922(26.9)	
Almost every day	512	2.9	313(61.1)	199(38.9)	
**Frequency of reading Newspaper**					1.672 (3), *p* = 0.643
Not at all	15,230	85.9	11,375(74.7)	3854(25.3)	
Less than once a week	1469	8.3	1061(72.2)	408(27.8)	
At least once a week	957	5.4	682(71.3)	275(28.7)	
Almost every day	75	0.4	44(59.3)	41(30.7)	
***Community level***					
**Residence**					3.303(1), *p* = 0.069
Urban	5260	30.0	3841(73.0)	1419(27.0)	
Rural	12,470	70.0	9322(74.8)	3149(25.2)	
**Wealth quintile**					49.061 (4), *p* < 0.001
Poorest	3573	20.2	2733(76.5)	840(23.5)	
Poorer	3927	22.1	3032(77.2)	895(22.8)	
Middle	3759	21.2	2778(73.9)	981(26.1)	
Richer	3414	19.3	2411(70.6)	1003(29.4)	
Richest	3057	17.2	2209(72.2)	848(27.8)	
**Family head**					43.544 (1), *p* < 0.001
Male	15,517	87.5	11,653(75.1)	3864(24.9)	
Female	2214	12.5	1511(68.2)	703(31.8)	
**Community literacy level**					31.404 (1), *p* < 0.001
Low	5611	31.6	4096(72.9)	1515(27.1)	
Medium	6575	37.1	4898(74.5)	1677(25.5)	
High	5,5 45	31.3	4186(75.5)	1359(24.5)	
**Community socio-economic status**					72.543(1), *p* < 0.001
Low	6823	38.5	4885(71.6)	1938(28.4)	
Medium	5684	32.1	4434(78.0)	1250(22.0)	
High	5224	29.5	3861(73.9)	1363(26.1)	

χ^2^ = Chi-square, *df* degree of freedom

### Measures of association (fixed effects)

Table 3 reports findings of the multi-level binary logistic regression analysis. From the final model (model 5), women aged 30–34 were more likely to use ITN compared with women in 45–49 age category [aOR = 1.11; Crl = 1.18–1.60]. Women with no education had higher odds of sleeping under ITN relative to highly educated women [aOR = 1.15; Crl = 1.03–2.10]. Compared to women who did not want their pregnancies at all, women who wanted their pregnancies [aOR = 1.12; Crl = 1.03–1.22] were more probable to use ITN. Women who were not subscribed to health insurance were less probable to use ITN relative to those who were subscribed [aOR = 0.77; Crl = 0.63–0.88]. Women who reported that they do not listen to radio at all had less odds of using ITN compared with women who listened to radio almost every day [aOR = 0.92; Crl = 0.77–0.96]. On the contrary, women who were not watching television (T.V.) at all were more probable to use ITN compared with those who watched T.V. almost every day [aOR = 1.29; Crl = 1.12–1.32]. Relative to rural women, women of urban residence were more likely to use ITN [aOR = 1.21; Crl = 1.14–1.30]. Poorest women were less probable to use ITN relative to richest women [aOR = 0.68; Crl = 0.72–0.90]. Women from male headed families revealed higher likelihood of ITN use relative to women from female-headed households [aOR = 1.31; Crl = 1.29–1.42]. Higher odds of ITN use was observed in communities with high literacy [aOR = 2.26; Crl = 2.11–3.20] and socio-economic level [aOR = 2.28; Crl = 2.12–3.39]. ITN use was highly probable for women from West Africa compared with women from Central Africa [aOR = 19.51; Crl = 5.19–33.04].

**Table 3:** Effect of individual, community and societal level factors on ITN utilisation among pregnant women

	Model 1		Model 2		Model 3		Model 4		Model 5	
	Empty Model		OR	[95% Crl]	OR	[95% Crl]	OR	[95% Crl]	aOR	[95% Crl]
**Fixed effect**										
***Individual level***										
**Age, years**										
15–19			0.85	[0.30–0.23]					0.84	[0.67–1.24]
20–24			1.16	[0.88–1.53]					1.18	[0.84–1.30]
25–29			1.14	[0.87–1.50]					2.02	[0.81–3.01]
30–34			1.14*	[1.06–1.49]					1.11**	[1.18–1.60]
35–39			1.08	[0.82–1.43]					1.14	[0.79–1.51]
40–44			1.06	[0.79–1.42]					2.09	[0.66–2.39]
45–49			1	[1]					1	[1]
**Education**										
No education			1.13	[0.95–1.34]					1.15*	[1.03–2.10]
Primary			1.13	[0.96–1.34]					1.17*	[1.07–1.32]
Secondary			1.09	[0.93–1.28]					1.08	[0.91–1.28]
Higher			1	[1]					1	[1]
**Marital Status**										
Married			1.08	[0.99–1.17]					1.09	[0.99–1.27]
Cohabiting			1	[1]					1	[1]
**Health Decision Making**										
Alone			1.20	[0.88–1.63]					1.21	[0.78–1.72]
With Partner			1.31	[0.97–1.77]					1.31	[0.91–1.80]
Husband/Partner Alone			1.35*	[1.02–1.82]					1.37	[0.94–1.69]
Other person			1	[1]					1	[1]
**Wanted current pregnancy**										
Wanted			1.06	[0.95–1.20]					1.12**	[1.03–1.22]
Later			1.07	[0.94–1.21]					1.09*	[1.04–1.18]
Not at all			1	[1]					1	[1]
**Health Insurance**										
No			0.75***	[0.66–0.85]					0.77***	[0.63–0.88]
Yes			1	[1]					1	[1]
**Frequency of listening to radio**										
Not at all			0.93	[0.76–1.14]					0.92*	[0.77–0.96]
Less than once a week			1.02	[0.84–1.26]					1.04	[0.83–1.30]
At least once a week			1.12	[0.92–1.37]					1.14	[0.94–1.41]
Almost every day			1	[1]					1	[1]
**Frequency of watching T.V**										
Not at all			1.33**	[1.08–1.64]					1.29*	[1.12–1.32]
Less than once a week			1.15	[0.92–1.44]					1.18	[0.91–1.46]
At least once a week			1.14	[0.93–1.42]					1.20	[0.91–1.48]
Almost every day			1	[1]					1	[1]
**Frequency of reading Newspaper**										
Not at all			0.97	[0.63–1.50]					0.99	[0.67–1.48]
Less than once a week			1.04	[0.67–1.61]					1.09	[0.81–1.88]
At least once a week			1.10	[0.71–1.71]					1.14	[0.75–1.81]
Almost every day			1	[1]					1	[1]
***Community level***										
**Residence**										
Urban					1.02	[0.96–1.09]			1.21**	[1.14–1.30]
Rural					1	[1]			1	[1]
**Wealth quintile**										
Poorest									0.68***	[0.72–0.90]
Poorer									1.08	[0.93–1.16]
Middle									1.09	[0.89–1.14]
Richer									1.04	[0.85–1.06]
Richest									1	[1]
**Family head**										
Male					1.31***	[1.21–1.41]			1.31***	[1.29–1.42]
Female					1	[1]			1	[1]
**Community literacy level**										
Low					1	[1]			1	[1]
Medium					1.17	[0.94–1.21]			1.21	[0.92–1.29]
High					2.11**	[1.99–3.21]			2.26**	[2.11–3.20]
**Community socio-economic status**										
Low					1	[1]			1	[1]
Medium					1.72*	[1.40–1.91]			1.75*	[1.40–2.22]
High					2.22**	[2.05–3.24]			2.28**	[2.12–3.39]
***Societal level***										
**Geographical location in SSA**										
Western							16.45***	[3.67–73.92]	19.51***	[5.19–33.04]
Eastern							13.91***	[3.46–55.95]	15. 69***	[4.70–30.19]
Central							14.56***	[3.65–58.10]	16.13***	[3.81–29.99]
Southern							1	[1]	1	[1]
**Random-effect**										
**Societal factors**										
Variance (95% CrI)	1.54	[1.21–1.87]	1.56	[1.22–1.91]	1.55	[1.24–1.89]	1.45	[1.17–1.64]	1.51	[1.15–1. 71]
VPC %, (95% Crl)	38.05	[25.60–49.20]	33.40	[21.80–39.80]	22.89	[15.64–29.1]	30.9	[21.50–44.60]	39.70	[34.01–43.05]
MOR (95% Crl)	2.02	[1.55–2.43]	2.04	[1.56–2.48]	2.10	[1.55–2.42]	2.90	[2.48–3.14]	4.47	[3.53–4.97]
Explained variation (%)	1.00	[1]	33.7	[31.8–39.5]	24.43	[19.8–30.1]	15.2	[14.30–15.80]	24.09	[21.40–28.90]
**Community level**										
Variance (95% CrI)	0.33	[0.28–0.38]	0.33	[0.28–0.39]	0.34	[0.27–0.38]	0.38	[0.32–0.44]	0.46	[0.35–0.49]
VPC %(95% Crl)	20.90	[13.0–27.5]	20.70	[12.7–27.4]	20.71	[17.41–29.34]	18.21	[14.80–26.41]	19.74	[13.7–25.22]
MOR (95% CrI)	1.73	[1.66–1.80]	1.73	[1.64–1.80]	1.81	[1.62–1.72]	1.92	[1.21–2.05]	1.81	[1.69–1.88]
Explained variation (%)	1.00	[1]	45.30	[41.5–50.9]	33.6	[28.7–38.9]	28.92	[22.41–33.82]	65.4	[59.2–72.1]
**Model fit statistics**										
Bayesian DIC	33,666		33,449		34,590		33,355		35,765	
**Sample size**										
Societal level	21		21		21		21		21	
Community level	9350		9350		9350		9350		9350	
Individual level	25,473		25,473		25,473		25,473		25,473	

*OR* Odds Ratio, *CrI* Credible Interval, *VPC* Variance Partition Coefficient, *MOR* Median Odds Ratio; 1 = reference; *p < 0.05, **p < 0.01, ***p < 0.001]

### Measures of variation (random effects)

The findings from the random effect is also presented in Table 3. From the empty or first model, there is a significant variation in ITN use across the geographical location of SSA countries [σ^2^ = 1.54; Crl = 1.21–1.87]. The variance partition coefficient (VPC) revealed that 38.05% [Crl = 25.60–49.20] variation in the use of ITN is attributable to societal factors whilst 20.90% [Crl = 13.0–27.5] variation is attributable to community level factors. The median odds ratios (MORs) also revealed that when a woman moves to a different society with higher chances of ITN use, she has 1.81 chances of using ITN.

## Discussion

The aim of this study was to investigate the individual, community and societal factors that affect ITN use in SSA. Beyond individual level, the study showed significant variation in ITN use across the community and geographical spheres. This highlights the significant role of community and societal environment in ITN use across SSA. The community in which a woman lives (e.g. residence and family head) cannot be under-estimated by any nation that seeks to ensure that all pregnant women utilise ITN [8, 56]. As such, instituting multi-sectoral collaboration between local and health authorities, public and private sectors whenever designing interventions aimed at achieving high ITN use among pregnant women is paramount [57]. Education on ITN use, also need to be contextually relevant to gain high acceptance [58].

The VPCs and MORs revealed that community and societal factors greatly affect ITN use. This emphasises the essence of ITN interventions to strategise beyond individual level factors and target issues at the community and society levels. That is, instead of targeting individual characteristics, community and society characteristics must be of interest as well. This can help in developing robust and tailored interventions that can bring about significant attitudinal change towards ITN. These findings are in consonance with previous evidence that have been documented within SSA and other parts of the world [59–62].

Geographically, women in Western and Central Africa had higher odds of ITN use. Hitherto, a number of studies have established the significant role of geographical variation in malaria and subsequent ITN use. For instance, in SSA, the WHO notes the highest exposure to malaria in pregnancy occur in Western and Central Africa (35% each) whilst 20% exposure occurs in Eastern and Southern Africa [1]. This report by the WHO is an indication that pregnant women in high-risk locations are more likely to use ITN compared to women in relatively lower risk locations of SSA. In as much as this is anticipated, much more concerted effort is required to achieve universal coverage. The findings reinforce the need for SSA governments and anti-malaria partner organisations to be conscious of geographical variation within SSA [63]. Being conscious of geographical variation could guide ITN mass media educational initiatives in distribution and advocacy.

Women aged 30–34 were more likely to use ITN compared with women in 45–49 age category. Compared with women aged 30–34, those in the 45–49 cohort may have multiple pregnancy experiences. It is possible that those aged 45–49 did not utilise ITN in some of their previous pregnancies, if not all of their previous pregnancies. If they had a malaria free pregnancy and childbirths in the past without necessarily using ITN, they can ignore ITN use in subsequent pregnancies. On this account, women aged 30–34 are expected to have high likelihood of ITN use because some of them may be experiencing their first pregnancies and would be much committed to ITN to protect themselves and fetus.

Poorest women were less probable to use ITN relative to richest women. This finding is contrary to earlier reports from some SSA countries where lower likelihood of ITN use has been noted among rich women relative to poorest/poor women [40, 64]. Since all women who participated in this study had ITNs, this finding may reflect pressure exuded by poverty and limited malaria/ITN education or knowledge possessed by the poorest women relative to richest women. Intensified education among poorest women is crucial because they may have ITNs but may be using it for other purposes or even sell them to make a living due non-availability of food and other essential necessities of their families [65]. The finding may reflect Maslow’s Hierarchy of Needs whereby the basic physiological needs such as food and accommodation are prioritised and satisfied before the complex ones such as safety and self-actualisation [66].

Compared to women who did not want their pregnancies at all, women who wanted their pregnancies were more probable to use ITN. Women who become pregnant at the desirable time (planned) initiate have early and consistent ANC visits, adhere to health precautions and are health conscious [67]. Conversely, women whose pregnancies occurred later or earlier than planned (unintended) undergo heightened stress levels, which attenuates their prospects of undertaking healthy measures such as sleeping under ITNs [66]. This underscores the need to have an integrated maternal health care system that concurrently caters for pregnancy intention (family planning services) and ITN use across SSA.

Women who were not subscribed to health insurance were less probable to use ITN relative to those who had subscribed. To subscribe to health insurance is an indication of one’s consciousness and readiness to enjoy good health [67]. By inference, women who are subscribed to health insurance are sensitive to health issues and more conscious about the demerits of not using ITN compared with those who are not subscribed [68]. Moreover, most SSA countries adopt health facilities as ITN distribution outlets [69]. Due to this, pregnant women who are enrolled to health insurance are more probable to frequent these health facilities for healthcare utilisation and inadvertently obtain regular reminders about the need to always sleep under the ITN [70].

Women who reported that they do not listen to radio at all had less odds of using ITN compared to women who listened to radio almost every day. On the contrary, women who were not watching television (T.V.) at all were more probable to use ITN compared with those watched T.V. almost every day. Generally, the mass media, especially radio has been an instrumental channel for ITN utilisation campaigns [10, 71] in addition to being the most impactful information outlet in Africa [72–74]. The influence of radio and T. V on ITN use as noticed in this study signals the need for careful selection of suitable information channels that can target masses of SSA populace. Thus, making radio the principal outlet whilst considering T. V and other mass media options as subsidiaries will be beneficial and impactful.

Relative to rural women, women of urban residence were more likely to use ITN. It is well established in the literature that ownership of ITN does not necessarily translate into utilisation. As purported by the Health Belief Model (HBM), the likelihood of taking an initiative to protect one’s health (i.e. ITN use in the context of this study) is dependent on self-efficacy, perceived susceptibility and cues to action, i.e. triggers/compelling motivations [75]. Given that most health care facilities in SSA are predominantly concentrated in urban locations [75, 76], women in urban settings stand a higher chances of being prompted and motivated to use their ITN [77].

Higher odds of ITN use was observed in communities with high literacy and socio-economic level. High literacy and high socio-economic status noted to boost empowerment status [43, 78]. Empowered persons or communities are likely to initiate measures that can enhance their health and holistic wellbeing. As a result, these findings are anticipated. This suggest that ITN interventions should incorporate measures that can enhance literacy and socio-economic status of communities and persons.

Women from male-headed households revealed higher likelihood of ITN use relative to women from female-headed households. This finding concurs with the observation from a Cameroon based cross-sectional study where Fokam, Kindzeka [79] reported that having a male household head positively impacted ITN use. There is therefore an indication that active involvement of men in ITN initiatives at the household level can create some leverage and help improve ITN use among pregnant women. Such an approach will be more imperative in SSA as most households and families are headed by men [80].

## Strengths and limitation

The cross-sectional design does not allow causal inference to be drawn between ITN and associated factors. There is the possibility of social desirability bias depending on the preferences of a woman’s household and surrounding circumstances during the survey. In all these, the study possesses some inherent strengths worth mentioning. Findings and recommendations from the study are supported by large and current national level datasets that reflect current ITN use across SSA. The study offers credible and generalizable information on the role of individual, community and societal level factors in ITN use in SSA.

## Conclusion

The study has revealed that in addition to individual level factors, community and societal factors (e.g. geography) affect ITN use in SSA. In as much as the study points towards the need to incorporate community and societal variations in ITN interventions, active involvement of men can yield better outcome for ITN utilisation interventions in SSA. A longitudinal study that ensures follow-up of pregnant women at regular interval may be required to establish consistency in use whilst a qualitative study may also help to better understand why some pregnant women may have ITN and refuse to use it.

## Data Availability

The datasets supporting the conclusions of this article are available in the Measure DHS repository, https://dhsprogram.com/data/available-datasets.cfm.

## Abbreviations

ANC: Antenatal Care
aOR: Adjusted Odds Ratio
Crl: Credible Interval
DHS: Demographic and Health Survey
HBM: Health Belief Model
ICF: Inner-City Fund
ITN: Insecticide Treated Net
MCMC: Markov Chain Monte Carlo
MOR: Median odds ratio
OR: Odds ratio
SSA: sub-Saharan Africa
T.V: Television
VIF: Variance inflation factor
VPC: Variance partition coefficient
WHO: World Health Organisation

## References

[1] WHO (2019). World malaria report 2019.

[2] WHO. Global technical strategy for malaria 2016–2030. Geneva; 2015. Available from: https://www.who.int/malaria/areas/global_technical_strategy/en.

[3] Centers for Disease Control and Prevention (2019). Treatment of Malaria: Guidelines for clinicians (United States).

[4] WHO (2017). Malaria in pregnant women.

[5] Lassi ZS, Mallick D, Das JK, Mal L, Salam RA, Bhutta ZA (2014). Essential interventions for child health. Reprod Health.

[6] Gamble C, Ekwaru J, ter Kuile F (2009). Insecticide-treated nets for preventing malaria in pregnancy. Cochrane Database Syst Rev.

[7] WHO. High burden to high impact: a targeted malaria response. Geneva; 2019. [cited 2020 June 6, 2020]. Available from: https://www.who.int/malaria/publications/atoz/high-impact-response/en/.

[8] Steketee RW, Parise ME (2001). The burden of malaria in pregnancy in malaria-endemic areas. Am J Trop Med Hyg.

[9] Adedokun ST, Uthman OA (2020). Individual and contextual correlates of mosquito net use among women in Nigeria. Malar J.

[10] Ankomah A, Adebayo SB, Arogundade ED, Anyanti J, Nwokolo E, Inyang U (2014). The effect of mass media campaign on the use of insecticide-treated bed nets among pregnant women in Nigeria. Malaria Res Treat.

[11] Dionne-Odom J, Westfall AO, Apinjoh TO, Anchang-Kimbi J, Achidi EA, Tita AT (2017). Predictors of the use of interventions to prevent malaria in pregnancy in Cameroon. Malar J.

[12] Leonard N, Eric FB, Judith AK, Samuel W (2016). Factors associated to the use of insecticide treated nets and intermittent preventive treatment for malaria control during pregnancy in Cameroon. Arch Public Health.

[13] WHO, UNICEF (2015). Achieving the MDG Malaria Target.

[14] Singh M, Brown G, Rogerson S (2013). Ownership and use of insecticide-treated nets during pregnancy in sub-Saharan Africa: a review. Malar J.

[15] van Eijk AM, Hill J, Alegana VA, Kirui V, Gething PW, ter Kuile FO, et al. Coverage of malaria protection in pregnant women in sub-Saharan Africa: a synthesis and analysis of national survey data. Lancet Infect Dis. 2011;11(3):190–207. 10.1016/S1473-3099(10)70295-4. 10.1016/S1473-3099(10)70295-4 PMC3119932 21273130

[16] Tapon F (2021). Defining Sub-Saharan Africa and the countries in it.

[17] Corsi DJ, Neuman M, Finlay JE, Subramanian S (2012). Demographic and health surveys: a profile. Int J Epidemiol.

[18] The DHS Program. DHS Overview. n.d.. Available from: https://dhsprogram.com/what-we-do/survey-Types/dHs.cfm.

[19] Zimbabwe National Statistics Agency and ICF International (2016). Zimbabwe demographic and health survey 2015: final report.

[20] Ministère de la Planification, du Développement et de l’Aménagement du Territoire - MPDAT/Togo, Ministère de la Santé - MS/Togo and ICF International (2015). Enquête Démographique et de Santé au Togo 2013–2014.

[21] Liberia Institute of Statistics and Geo-Information Services - LISGIS, Ministry of Health and Social Welfare/Liberia, National AIDS Control Program/Liberia, and ICF International (2014). Liberia demographic and health survey 2013.

[22] Zambia Statistics Agency, Ministry of Health (MOH) Zambia, and ICF (2019). Zambia demographic and health survey 2018.

[23] Ministerio da Saude - MISAU/Moçambique, Instituto Nacional de Estatística - INE/Moçambique and ICF International (2015). Moçambique Inquérito Demográfico e de Saúde.

[24] National Statistical Office - NSO/Malawi and ICF (2017). Malawi demographic and health survey 2015–16.

[25] National Population Commission - NPC/Nigeria and ICF (2019). Nigeria Demographic and Health Survey 2018.

[26] Ministère du Plan et Suivi de la Mise en œuvre de la Révolution de la Modernité - MPSMRM/Congo, Ministère de la Santé Publique - MSP/Congo and ICF International (2014). Enquête Démographique et de Santé en République Démocratique du Congo 2013–2014.

[27] National Institute of Population Research and Training (NIPORT), and ICF (2020). Bangladesh demographic and health survey 2017-18.

[28] Statistics Sierra Leone - SSL and ICF International (2014). Sierra Leone demographic and health survey 2013.

[29] The Nambia Ministry of Health and Social Services - MoHSS - and ICF International (2014). The Namibia demographic and health survey 2013.

[30] Institut National de la Statistique - INS/Niger and ICF International (2013). Enquête Démographique et de Santé et à Indicateurs Multiples du Niger 2012.

[31] Uganda Bureau of Statistics - UBOS and ICF (2018). Uganda demographic and health survey 2016.

[32] Direction Générale de la Statistique - DGS/Gabon and ICF International (2013). Enquête Démographique et de Santé du Gabon 2012.

[33] Ghana Statistical Service (GSS), Ghana Health Service (GHS), ICF International. Ghana Demographic and Health Survey 2014. Accra: Statistical Service; 2015.

[34] Ministère à la Présidence chargé de la Bonne Gouvernance et du Plan Burundi - MPBGP, Ministère de la Santé Publique et de la Lutte contre le Sida Burundi - MSPLS, Institut de Statistiques et d’Études Économiques du Burundi - ISTEEBU, et ICF (2017). Troisième Enquête Démographique et de Santé au Burundi.

[35] Ministry of Health, Community Development, Gender, Elderly and Children - MoHCDGEC/Tanzania Mainland, Ministry of Health - MoH/Zanzibar, National Bureau of Statistics - NBS/Tanzania, Office of Chief Government Statistician - OCGS/Zanzibar, and ICF (2016). Tanzania demographic and health survey and malaria Indicator survey (TDHS-MIS) 2015-16.

[36] Kenya National Bureau of Statistics, Ministry of Health/Kenya, National AIDS Control Council/Kenya, Kenya Medical Research Institute, National Council for Population and Development/Kenya, and ICF International (2015). Kenya demographic and health survey 2014.

[37] National Institute of Statistics and Economic Analysis (INSAE) and ICF. Demographic and health survey and with multiple indicators (EDS-MICS 2014–2015). Rockville: INSEED, MSP et ICF International; 2015. (16) (PDF) Predictors of Female Genital Mutilation/Cutting among Daughters of Women aged 15–49 in Guinea: A Multilevel Analysis of the 2018 Demographic and Health Survey Data. Available from: https://www.researchgate.net/publication/349849213_Predictors_of_Female_Genital_MutilationCutting_among_Daughters_of_Women_aged_15-49_in_Guinea_A_Multilevel_Analysis_of_the_2018_Demographic_and_Health_Survey_Data Accessed 14 Mar 2021.

[38] Cellule de Planification et de Statistique, Institut National de la Statistique, Centre d’Études et d’Information Statistiques, I. C. F. International. Mali Enquête Démographique et de Santé (EDSM V) 2012-2013; 2014. Available from http://dhsprogram.com/pubs/pdf/FR286/FR286.pdf. Accessed 14 Mar 2021.

[39] MEASURE Evaluation, MEASURE DHS, President’s Malaria Initiative, Roll Back Malaria Partnership, UNICEF, WHO (2013). Household survey indicators for malaria control.

[40] Olapeju B, Choiriyyah I, Lynch M, Acosta A, Blaufuss S, Filemyr E, et al. Age and gender trends in insecticide-treated net use in sub-Saharan Africa: a multi-country analysis. Malar J. 2018;17(1):423. 10.1186/s12936-018-2575-z. 10.1186/s12936-018-2575-z PMC6234545 30428916

[41] Mbengue MAS, Bei AK, Mboup A, Ahouidi A, Sarr M, Mboup S, et al. Factors influencing the use of malaria prevention strategies by women in Senegal: a cross-sectional study. Malar J. 2017;16(1):470. 10.1186/s12936-017-2095-2. 10.1186/s12936-017-2095-2 PMC5697112 29157243

[42] Jacobs C, Moshabela M, Maswenyeho S, Lambo N, Michelo C (2017). Predictors of antenatal care, skilled birth attendance, and postnatal care utilization among the remote and poorest rural communities of Zambia: a multilevel analysis. Front Public Health.

[43] Yaya S, Uthman OA, Ekholuenetale M, Bishwajit G (2018). Women empowerment as an enabling factor of contraceptive use in sub-Saharan Africa: a multilevel analysis of cross-sectional surveys of 32 countries. Reprod Health.

[44] Seidu AA, Darteh EK, Agbaglo E, Dadzie LK, Ahinkorah BO, Ameyaw EK, et al. Barriers to accessing healthcare among women in Ghana: a multilevel modelling. BMC Public Health. 2020;20(1):1–2. 10.1186/s12889-020-10017-8 PMC7745480 33334326

[45] Øvretveit J (2011). Understanding the conditions for improvement: research to discover which context influences affect improvement success. BMJ Qual Saf.

[46] Nkuo-Akenji T, Ntonifor NN, Ndukum MB, Kimbi HK, Abongwa EL, Nkwescheu A (2006). Environmental factors affecting malaria parasite prevalence in rural Bolifamba, south-West Cameroon. Afr J Health Sci.

[47] Endo N, Eltahir EA (2016). Environmental determinants of malaria transmission in African villages. Malaria J.

[48] Mohammadkhani M, Khanjani N, Bakhtiari B, Sheikhzadeh K (2016). The relation between climatic factors and malaria incidence in Kerman, south east of Iran. Parasite Epidemiol Contr.

[49] United Nations (1999). Standard country or area codes for statistics use, 1999 (Revision 4).

[50] Rasbash J, Charlton C, Jones K, Pillinger R (2014). Manual supplement for MLwiN version 2.31.

[51] Curran JM (2005). An introduction to Bayesian credible intervals for sampling error in DNA profiles. Law Probabil Risk.

[52] Merlo J, Chaix B, Yang M, Lynch J, Råstam L (2005). A brief conceptual tutorial of multilevel analysis in social epidemiology: linking the statistical concept of clustering to the idea of contextual phenomenon. J Epidemiol Commun Health.

[53] Larsen K, Merlo J (2005). Appropriate assessment of neighborhood effects on individual health: integrating random and fixed effects in multilevel logistic regression. Am J Epidemiol.

[54] Akinwande MO, Dikko HG, Samson A (2015). Variance inflation factor: as a condition for the inclusion of suppressor variable (s) in regression analysis. Open J Stat.

[55] The DHS Program (n.d.). Protecting the privacy of DHS survey respondents. Retrieved from https://dhsprogram.com/methodology/Protecting-the-Privacy-of-DHS-Survey-Respondents.cfm on March 13, 2021.

[56] Hill J, Hoyt J, van Eijk AM, D'Mello-Guyett L, ter Kuile FO, Steketee R (2013). Factors affecting the delivery, access, and use of interventions to prevent malaria in pregnancy in sub-Saharan Africa: a systematic review and meta-analysis. PLoS Med.

[57] Dhewantara PW, Ipa M, Widawati M (2019). Individual and contextual factors predicting self-reported malaria among adults in eastern Indonesia: findings from Indonesian community-based survey. Malaria J.

[58] Aberese-Ako M, Magnussen P, Ampofo GD, Tagbor H (2019). Health system, socio-cultural, economic, environmental and individual factors influencing bed net use in the prevention of malaria in pregnancy in two Ghanaian regions. Malaria J.

[59] Mensah EA, Anto F (2020). Individual and community factors associated with household insecticide-treated Bednet usage in the Sunyani West District of Ghana two years after mass distribution. J Environ Public Health.

[60] Ruyange MM, Condo J, Karema C, Binagwaho A, Rukundo A, Muyirukazi Y (2016). Factors associated with the non-use of insecticide-treated nets in Rwandan children. Malar J.

[61] Admasie A, Zemba A, Paulos W (2018). Insecticide-treated nets utilization and associated factors among under-5 years old children in Mirab-Abaya District, Gamo-Gofa zone, Ethiopa. Front Public Health.

[62] Nkumama IN, O’Meara WP, Osier FH (2017). Changes in malaria epidemiology in Africa and new challenges for elimination. Trends Parasitol.

[63] Ricotta E, Oppong S, Yukich JO, Briët OJ (2019). Determinants of bed net use conditional on access in population surveys in Ghana. Malar J.

[64] Berthe S, Harvey SA, Lynch M, Koenker H, Jumbe V, Kaunda-Khangamwa B, et al. Poverty and food security: drivers of insecticide-treated mosquito net misuse in Malawi. Malar J. 2019;18(1):320. 10.1186/s12936-019-2952-2. 10.1186/s12936-019-2952-2 PMC6751583 31533727

[65] Maslow AH (1943). A theory of human motivation. Psychol Rev.

[66] Dibaba Y, Fantahun M, Hindin MJ (2013). The effects of pregnancy intention on the use of antenatal care services: systematic review and meta-analysis. Reprod Health.

[67] Thogarapalli N, Mkandawire P, Rulisa S, Luginaah IJ (2015). Investigating the association between pregnancy intention and insecticide-treated bed net (ITN) use: a cross-sectional study of pregnant women in Rwanda. J Public Health.

[68] Rosenstock IM (1974). Historical origins of the health belief model. Health Educ Monogr.

[69] Binagwaho A, Hartwig R, Ingeri D, Makaka A (2012). Mutual health insurance and its contribution to improving child health in Rwanda. Passauer Diskussionspapiere-Volkswirtschaftliche Reihe.

[70] Skarbinski J, Mwandama D, Luka M, Jafali J, Wolkon A, Townes D, et al. Impact of health facility-based insecticide treated Bednet distribution in Malawi: Progress and challenges towards achieving universal coverage. PLoS One. 2011;6(7):e21995. 10.1371/journal.pone.0021995. 10.1371/journal.pone.0021995 PMC3141001 21811553

[71] BBC Monitoring (2016). Media guide Uganda.

[72] Myers M (2009). Radio and development in Africa: a concept paper.

[73] Africanews (2017). Radio is Africa’s most influential information outlet - UNESCO survey.

[74] Oladipo JA (2014). Utilization of health care services in rural and urban areas: a determinant factor in planning and managing health care delivery systems. Afr Health Sci.

[75] Dominic A, Ogundipe A, Ogundipe O. Determinants of women access to healthcare services in Sub-Saharan Africa. Open Public Health J. 2019;12(1):504–14.

[76] Oloyede O (2017). Rural-urban disparities in health and health care in Africa: cultural competence, lay-beliefs in narratives of diabetes among the rural poor in the eastern Cape Province of South Africa. Afr Sociol Rev.

[77] Alsop R, Heinsohn N. Measuring empowerment in practice: structuring analysis and framing indicators. Washington, DC: The World Bank; 2005.

[78] Santillan D, Schuler SR, Anh HT, Minh TH, Trang QT, Duc NM (2004). Developing indicators to assess women's empowerment in Vietnam. Dev Pract.

[79] Fokam EB, Kindzeka GF, Ngimuh L, Dzi KT, Wanji S (2017). Determination of the predictive factors of long-lasting insecticide-treated net ownership and utilisation in the Bamenda Health District of Cameroon. BMC Public Health.

[80] Farnworth CR, Colverson KE (2015). Building a gender-transformative extension and advisory facilitation system in Sub-Saharan Africa. J Gender Agric Food Sec.

